# Development of a bespoke phantom to optimize molecular PET imaging of pituitary tumors

**DOI:** 10.1186/s40658-023-00552-9

**Published:** 2023-06-01

**Authors:** Daniel Gillett, Daniel Marsden, Rosy Crawford, Safia Ballout, James MacFarlane, Merel van der Meulen, Bethany Gillett, Nick Bird, Sarah Heard, Andrew S. Powlson, Thomas Santarius, Richard Mannion, Angelos Kolias, Ines Harper, Iosif A. Mendichovszky, Luigi Aloj, Heok Cheow, Waiel Bashari, Olympia Koulouri, Mark Gurnell

**Affiliations:** 1 Department of Nuclear Medicine, Cambridge University Hospitals NHS Foundation Trust, Cambridge Biomedical Campus, Cambridge, CB2 0QQ UK; 2 Cambridge Endocrine Molecular Imaging Group, University of Cambridge and Cambridge University Hospitals NHS Foundation Trust, Cambridge Biomedical Campus, Cambridge, CB2 0QQ UK; 3 Clinical Engineering, Cambridge University Hospitals NHS Foundation Trust, Cambridge Biomedical Campus, Cambridge, CB2 0QQ UK; 4 East Anglian Regional Radiation Protection Service, Cambridge University Hospitals NHS Foundation Trust, Cambridge Biomedical Campus, Cambridge, CB2 0QQ UK; 5 Division of Neurosurgery, Department of Clinical Neurosciences, University of Cambridge and Cambridge University Hospitals NHS Foundation Trust, Cambridge Biomedical Campus, Cambridge, CB2 0QQ UK; 6 Department of Radiology, University of Cambridge and Cambridge University Hospitals NHS Foundation Trust, Cambridge Biomedical Campus, Cambridge, CB2 0QQ UK; 7 Metabolic Research Laboratories, Wellcome-MRC Institute of Metabolic Science University of Cambridge, National Institute for Health Research Cambridge Biomedical Research Centre, Addenbrooke’s Hospital, Hills Road, Cambridge, CB2 0QQ UK

**Keywords:** 3D printing radioactive phantom, Optimization of molecular PET imaging, Pituitary tumors/adenomas

## Abstract

**Background:**

Image optimization is a key step in clinical nuclear medicine, and phantoms play an essential role in this process. However, most phantoms do not accurately reflect the complexity of human anatomy, and this presents a particular challenge when imaging endocrine glands to detect small (often subcentimeter) tumors. To address this, we developed a novel phantom for optimization of positron emission tomography (PET) imaging of the human pituitary gland. Using radioactive 3D printing, phantoms were created which mimicked the distribution of ^11^C-methionine in normal pituitary tissue and in a small tumor embedded in the gland (i.e., with no inactive boundary, thereby reproducing the in vivo situation). In addition, an anatomical phantom, replicating key surrounding structures [based on computed tomography (CT) images from an actual patient], was created using material extrusion 3D printing with specialized filaments that approximated the attenuation properties of bone and soft tissue.

**Results:**

The phantom enabled us to replicate pituitary glands harboring tumors of varying sizes (2, 4 and 6 mm diameters) and differing radioactive concentrations (2 ×, 5 × and 8 × the normal gland). The anatomical phantom successfully approximated the attenuation properties of surrounding bone and soft tissue. Two iterative reconstruction algorithms [ordered subset expectation maximization (OSEM); Bayesian penalized likelihood (BPL)] with a range of reconstruction parameters (e.g., 3, 5, 7 and 9 OSEM iterations with 24 subsets; BPL regularization parameter (*β*) from 50 to 1000) were tested. Images were analyzed quantitatively and qualitatively by eight expert readers. Quantitatively, signal was the highest using BPL with *β* = 50; noise was the lowest using BPL with *β* = 1000; contrast was the highest using BPL with *β* = 100. The qualitative review found that accuracy and confidence were the highest when using BPL with *β* = 400.

**Conclusions:**

The development of a bespoke phantom has allowed the identification of optimal parameters for molecular pituitary imaging: BPL reconstruction with TOF, PSF correction and a *β* value of 400; in addition, for small (< 4 mm) tumors with low contrast (2:1 or 5:1), sensitivity may be improved using a *β* value of 100. Together, these findings should increase tumor detection and confidence in reporting scans.

**Supplementary Information:**

The online version contains supplementary material available at 10.1186/s40658-023-00552-9.

## Background

Optimization is a key aspect of any imaging pathway, and phantoms play an essential role in this process. In nuclear medicine, numerous phantoms are commercially available ranging from the 2D Williams' liver phantom (for gamma camera imaging) to the 3D Jaszczak phantom [for positron emission tomography (PET)] [1]. However, while these are useful, they do not accurately reflect the complexity of human anatomy. Therefore, a number of anthropomorphic phantoms have been developed to facilitate optimization in specific contexts, including brain imaging (e.g., Hoffman brain phantom; striatal DAT scan phantom) and myocardial perfusion imaging [2, 3]. However, when optimization is required but there is no commercially available resource for the tissue/organ of interest, then fabrication of a bespoke phantom can be considered. This may involve various approaches, ranging from the use of syringes filled with radioactivity [4] to more advanced techniques such as 3D printing [5]. In nuclear medicine, 3D printing can be used to create voids which can then be filled with radioactive material or, more recently, we and others have developed techniques that allow direct 3D printing of radioactive objects [6–8]. This latter technique has the advantage of creating precisely shaped objects that are radioactive. Active objects can be positioned next to each other without an inactive boundary, which would otherwise create an artificial area of zero counts. This consideration is especially important when the object of interest may be very small and embedded in another structure, as may be the case with small pituitary tumors [pituitary adenomas (PA)] that lie hidden within the normal pituitary gland. This highlights that even standard phantom objects that reasonably approximate the pituitary gland, such as the 10-mm sphere of NEMA PET image quality phantom, are inadequate for this type of image optimization because it is not possible to embed tumor-like objects within them.

Our group and others have successfully utilized ^11^C-Methionine PET/CT (Met-PET) to image PA where conventional anatomical imaging (MRI) has not been able to confidently identify the location of the adenoma [9, 10]. However, just like all PET imaging, Met-PET is limited by the spatial resolution and sensitivity of the scanner and the reconstruction algorithm including the corrections applied. These limitations mean that small adenomas (< 10 mm) and those with low uptake can be difficult to detect [10, 11]. To minimize the effects of these limitations on the final images, optimization of the imaging protocol is crucial. However, currently, there is no commercially available option for PA imaging, and therefore, we set out to develop a bespoke phantom that could accurately depict both the normal gland and tumor. This required us to create a phantom that was pituitary-shaped, which could be imaged either with or without an adenoma-sized object embedded within it, with no inactive boundary. In addition, we considered it important to mimic as closely as possible the attenuation properties of the surrounding anatomical structures. To do this, we adopted a recently published method that uses material extrusion (MEX) 3D printing together with concrete-filled filament to create bone phantoms that have comparable Hounsfield units to bone seen on a computed tomography (CT) scan [12].

Using this phantom, we have examined iterative reconstruction algorithms for PET imaging such as ordered subset expectation maximization (OSEM) and Bayesian penalized likelihood (BPL) [13, 14]. Specifically, we have sought to optimize the parameters used in these algorithms, including the use of point spread function correction (PSF) and voxel size. For OSEM reconstruction we also explored post-reconstruction filter size and the number of iterative reconstruction updates, while for BPL, we examined the regularization parameter *β*. The use of point spread function correction has been shown many times to improve lesion detectability in PET imaging [4, 15, 16] but, to the best of our knowledge, it has never been tested in a small phantom that has two different radioactive concentrations in contact with each other, such as that mimicking a small PA (i.e., pituitary microadenoma) embedded within an otherwise normal pituitary gland. Finally, the number of iterative reconstruction updates can enhance signal peaks but, importantly, also increase image noise. This issue has been overcome by using BPL algorithms, which allow full convergence while at the same time limiting noise [17]. These reconstruction parameters have been optimized for other types of PET imaging but they may not be applicable to the pituitary gland. Therefore, here we report the methodology for creating a bespoke molecular imaging pituitary phantom and how it was used to optimize OSEM and BPL iterative reconstruction algorithms.

## Methods

### Phantom design

**Fig. 1:**
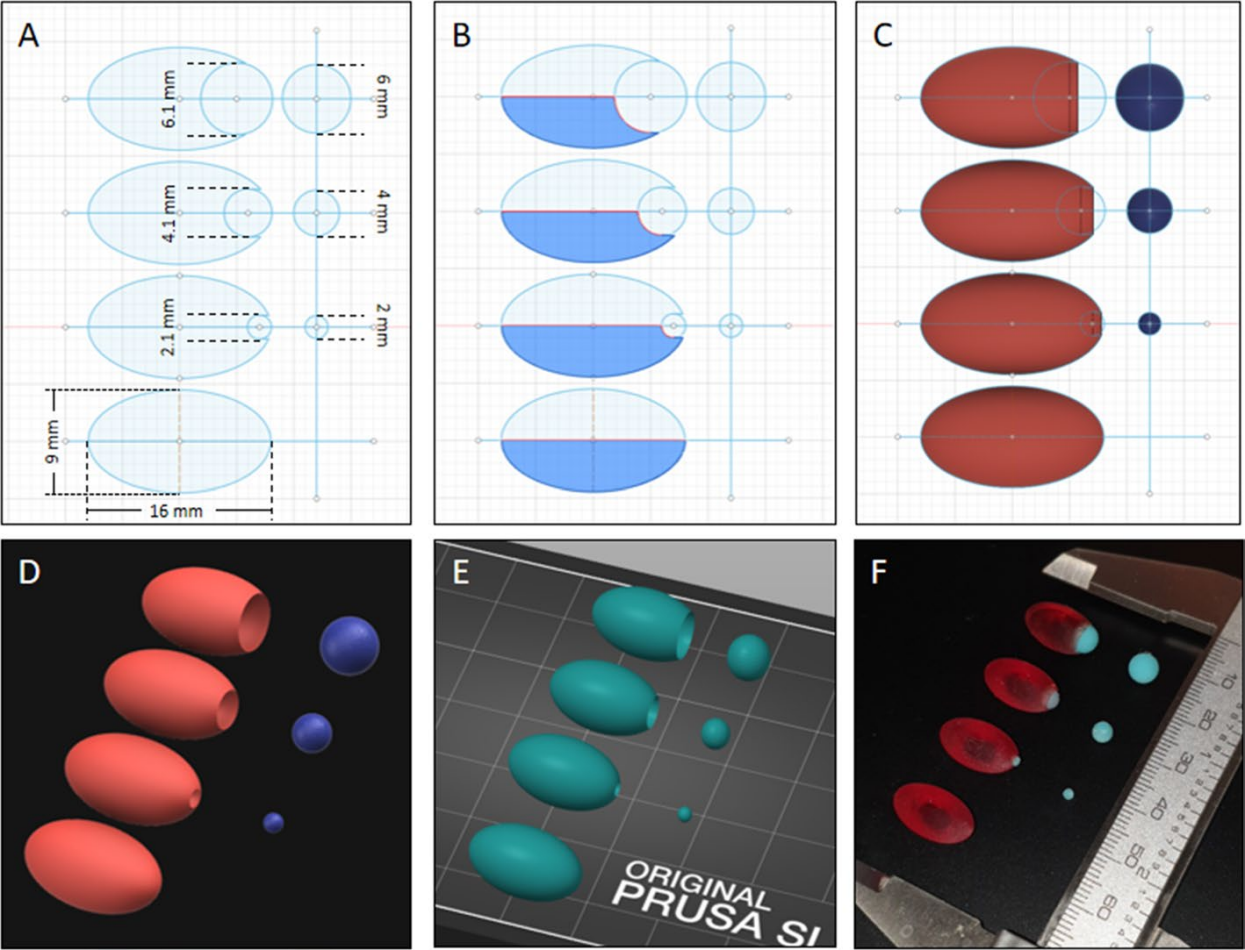
Design and 3D printing of pituitary gland phantom and associated tumors. CAD 2D sketch of pituitary gland design (**A**). The pituitary gland was approximated as an ellipse with a length of 16 mm and a height of 9 mm. The tumors were spherical with diameters of 2, 4 and 6 mm. So that the tumors could be embedded into the pituitary gland, three more designs were made with 2.1-, 4.1- and 6.1-mm spherical indentations. Using the revolve function, the face of the design (highlighted in blue) (**B**) was revolved around the horizontal axis to create 3D ellipsoids with and without indentations (**C**). The objects were exported as STL files (**D**) and loaded into PrusaSlicer to be prepared for printing (**E**). After printing the pituitary gland and tumors with different radioactive concentrations, these were combined to create pituitary glands containing tumors of different contrast (**F**)

The pituitary gland and pituitary tumors were printed using a previously described radioactive 3D printing methodology [6]. The pituitary gland was designed as an ellipsoid with a length of 16 mm and a height of 9 mm. Three spheres were designed to approximate tumors of 2, 4 and 6 mm diameters. To enable these spheres to be embedded in the pituitary phantom, indentations of 2.1, 4.1 and 6.1 mm were added to one of the pituitary gland designs. A margin of 0.1 mm was required so that the spheres would fit inside the printed indent. These phantoms were designed in a basic CAD software package as 2D sketches (Fig. 1A). These sketches were used to create 3D objects by selecting a face and an axis to pivot around (Fig. 1B). The resulting objects (Fig. 1C) were converted to high-resolution STL files (Fig. 1D) and uploaded into PrusaSlicer to prepare the objects for printing (Fig. 1E). The spheres were printed using radioactive concentrations that were different from the phantom normal gland and could then be combined to create a pseudo-pituitary gland with and without an embedded tumor (Fig. 1F). The activity concentrations were based on those seen in clinical practice. Typically, the cerebellum has an activity concentration of between 2.4 and 6.7 kBq/ml depending on the administered activity and the size of the patient. Based on this range, a target concentration of 5 kBq/ml was selected. The mean concentration in the pituitary gland region (including the tumors) can vary significantly depending on the size, extent and type of tumor [range between 1.0 (5.0 kBq/ml) and 10 (50 kBq/ml) times the cerebellum concentration]. A target of 20 kBq/ml was selected for the pituitary gland concentration.

**Fig. 2:**
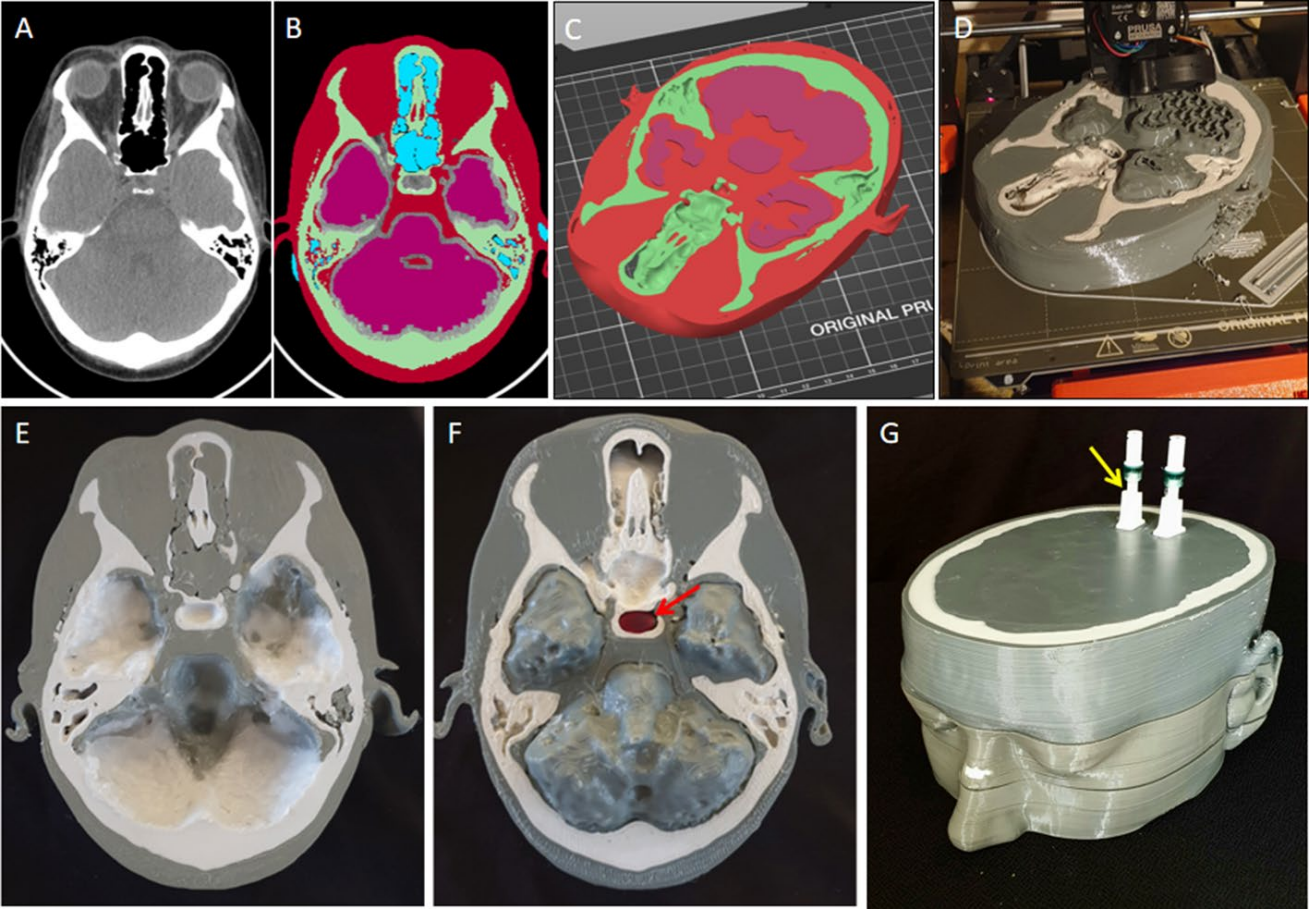
Development and printing of brain and skull phantom. The CT scan of a patient (**A**) was used to create the surrounding brain and skull phantom by segmenting the soft tissue (red), bone (green), air (blue) and brain (pink) (**B**). The segments were exported as STL files and loaded into PrusaSlicer (**C**) for printing (**D**). Following printing of the two halves (**E**, **F**), the radioactive pituitary gland ± tumor were inserted into the pituitary fossa (red arrow in panel **F**). Thereafter, the two halves were brought together (**G**) before filling with radioactivity via the ports (yellow arrow in panel **G**)

The surrounding phantom was based on CT and MRI images obtained from a patient who had undergone clinical imaging to localize a pituitary tumor (causing Cushing Disease). The patient provided informed written consent for their scans to be used to create this phantom. Using 3D Slicer [18] the patient’s CT scan was segmented into air, soft tissue and bone using the thresholding tool (Fig. 2A). The threshold ranges were set to − 1000 to − 300, − 301 to 100 and 101 to 1000 HU for the air, soft tissue and bone regions, respectively (Fig. 2B). In addition, areas of the brain in proximity to the pituitary fossa (including the temporal lobes), were printed as partially hollow structures to allow the addition of radioactivity to mimic background brain activity as observed in clinical scans. The air, bone and soft tissue regions (with the exception of the brain) were divided into two parts through the axial plane at the level of the pituitary fossa. In contrast, the brain was left whole and connected to the superior part of the phantom (see Fig. 2D, F). This allowed the brain segment to be filled as a single volume following printing. This required the inferior part of the phantom (Fig. 2E) to include a small margin [2 mm] around the brain (Fig. 2B) to accommodate the protruding brain segment from the superior part (Fig. 2F, G). Finally, a cavity was introduced to mimic the pituitary fossa and to accommodate the printed pituitary gland ± tumor (Fig. 2F). To enable the brain cavity to be filled with radioactivity, two ports were connected to the superior part of the phantom.

### Material extrusion 3D printing

Material extrusion 3D printing was performed using a Prusa Mk3s with the Multi-Material Unit 2 (MMU) with a 0.8-mm nozzle and a 0.4-mm layer height. Two materials were used to create the anatomical structures surrounding the pituitary gland. Soft tissue structures were printed with PETG filament with an infill of 10% gyroid pattern and 100% rectilinear pattern for the fillable brain segment and the surrounding soft tissue, respectively. The bones were printed using concrete-filled PLA filament (Concrete Stonefil, FormFuture) with an infill of 100% rectilinear pattern. Both materials were printed using a nozzle temperature of 230 °C and a bed temperature of 90℃. Using these settings, the inferior and superior parts of the phantom took 28 and 24 h to print, respectively.

### Radioactive 3D printing

Radioactive resin 3D printing is based on vat photopolymerization (VP) and was undertaken according to a previously reported method [6] which enables 3D printing resin to be labelled with ^18^F. Printing was carried out on a Prusa SL1 3D printer (Prusa Research, Prague, Czech Republic), using Prusa Tough resin (with different colors used to differentiate the radioactive concentrations), a layer height of 0.1 mm, with an initial exposure time of 35 s and subsequent layer exposure times of 6 s. Four radioactive concentrations (20, 40, 100 and 160 kBq/mL at the time of imaging) were created and used to generate contrast in the PET image. Pituitary glands with different sized indentations (see Fig. 1) were printed using the lowest concentration. Spheres (used to approximate pituitary tumors) of 2-, 4- and 6-mm diameter were printed with the three higher concentrations and inserted into the indents of the normal pituitary gland (see Fig. 1).

For each concentration, four cylinders were printed (diameter 8 mm, height 10 mm) together with the phantom objects and used to measure the true ratio between the concentrations. The activities of the cylinders were measured in an automated sample counter (Wallac Wizard 2480, Waltham, MA, USA) for 150 s using the ^18^F energy window (511 keV ± 20%). The counts per minute (CPM) were background and decay corrected before being used to calculate the ratios. The expected concentration ratios were 2:1, 5:1 and 8:1. The printing time was 24 min for all of the concentrations. The 5:1 6-mm tumor failed to print and therefore was not used in the study.

### Phantom assembly and imaging

To mimic the background brain activity concentration (5 kBq/ml) in a Met-PET scan, 3 MBq of ^18^F was added to the 600-ml brain cavity in the phantom (Fig. 2). The radioactive pituitary glands ± tumor were wrapped in a single layer of cling film to hold them together and minimize contamination. They were then inserted into the pituitary fossa on the phantom (Fig. 2F). The two sections of the brain phantom were then brought together and secured (with adhesive tape). The phantom was then positioned on the scanning table between a perspex cylinder (diameter: 15 cm; length: 20 cm) and perspex cuboid (width: 15 cm; height: 15 cm; length: 18 cm) to create scatter material similar to that observed in a patient. In addition, approximately 100 MBq in a glass vial was placed in a perspex cylinder (diameter: 25 cm; length: 30 cm) to approximate the activity from the torso of a patient. Imaging was performed using a Discovery 690 PET/CT scanner (GE Healthcare, Chicago, Illinois, United States). One 5-min bed position centered on the phantom’s pituitary gland was acquired for each phantom setup. A CT acquisition was acquired for each PET scan to correct them for attenuation. The CT scan was acquired with 140 kV, fixed mA of 220, a rotation speed of 0.5 s, a pitch of 0984:1, 30 cm field of view, a slice thickness of 1.25 mm and a 1.25-mm spacing interval. The CT was reconstructed using filtered back projection.

### Reconstruction

The OSEM and BPL iterative reconstruction algorithms were both available from GE (VPFX and Q.Clear, respectively). The parameters of these algorithms were varied and are summarized in Table 1. All reconstructions included attenuation correction (AC) and scatter correction (SC) and had a field of view (FOV) of 30 cm^2^. The size of the pixels were 2.34 mm × 2.34 mm and 1.17 mm × 1.17 mm for the 128 × 128 and 256 × 256 matrix sizes , respectively and will be referred to by the matrix sizes 128 and 256.

**Table 1:** Reconstruction parameters and options investigated

Parameter/correction	Algorithm	Options	Expert evaluation
Time of flight	OSEM, BPL*	Off and On	Not applicable
Point spread function correction (PSF)	OSEM, BPL*	Off and On	Off and On
Matrix size (with 30 cm^2^ FOV)	OSEM, BPL*	128 × 128 and 256 × 256	128 × 128 and 256 × 256
Post-reconstruction smoothing (Gaussian—full width half maximum [FWHM])	OSEM	1, 2 and 3 mm	2 mm
OSEM Iterations (all with 24 subsets)	OSEM	3, 5, 7 and 9	3, 5, 7 and 9
Regularization parameter (*β*)	BPL	50, 100, 200, 300, 400, 500, 600, 700, 800, 900 and 1000	50, 100, 200, 300, 400, 500, 600, 800 and 1000

*Parameter applicable to both OSEM and BPL but was only compared for OSEM. The optimal option was then included in BPL optimization

### CT image analysis

The patient’s CT scan that was used to create the anatomical part of the phantom was compared qualitatively and quantitatively to the CT scan of the phantom. The mean and standard deviation of the Hounsfield units was assessed in the bone and soft tissue regions.

Three-dimensional slicer was used to extract the information from the scans. The scans were registered together using anatomical landmarks (known as fiducial marker registration in 3D slicer) and then, using the phantom images, the bone and soft tissue volumes were segmented using the thresholding tool. The same volumes were applied to the patient scan after ensuring the volumes were correctly aligned.

### PET image quality analysis

Three quantitative metrics were used to assess the image quality: maximum signal, contrast, and noise. The maximum voxel value in the pituitary gland was identified and normalized to the signal in the cerebellum for consistency [19]. Using a line profile placed at the center of the gland, contrast was defined as the ratio of the maximum signals on each side of the midline. The line profiles were also used to qualitatively compare the different reconstructions. For the assessment of noise, the coefficient of variation (CoV) of the signal in the phantom cerebellum was used as a relative measure of noise in the pituitary gland. (Direct measurement of pituitary gland noise was not possible due to its small size.)

**Fig. 3:**
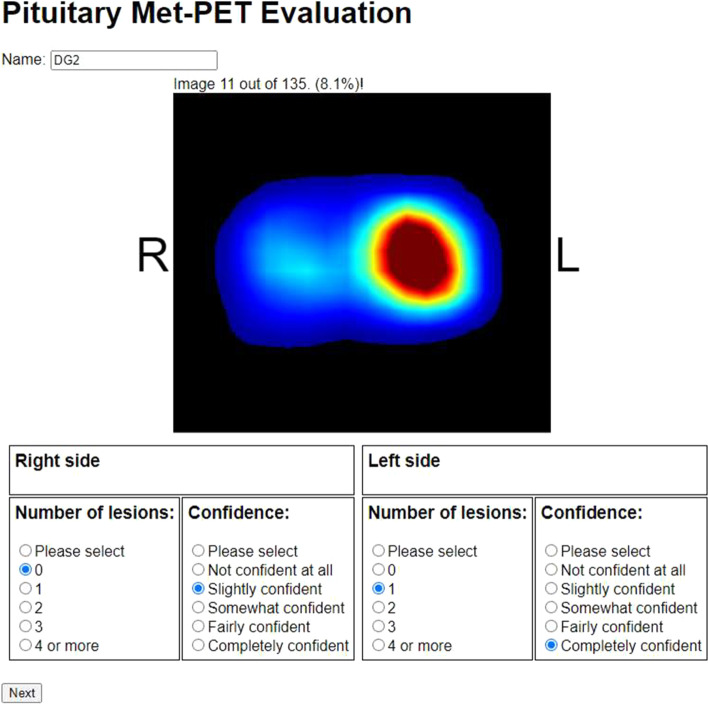
Screenshots of purpose-built online questionnaire tool. For each image, the user recorded how many lesions they could see on the right and left sides of the pituitary gland and their degrees of confidence. After submitting each evaluation, the next image was displayed; readers were unable to return to earlier images

To assess image quality, eight expert readers (one nuclear medicine physician, two radiologists with nuclear medicine experience, two neurosurgeons and three endocrinologists with expertise in molecular imaging) were asked how many lesions were evident (ranging between 0 and 4) in each side of the pituitary gland based on their review of individual PET datasets without a CT (Fig. 3). In addition, they were asked to indicate how confident they were in their assessment, using a Likert scale which ranged from not confident at all (score = 0) to completely confident (score = 4) (Fig. 3). Importantly, the readers were blinded to the reconstruction parameters used and had no knowledge that the images were of a phantom. After an initial review of the quantitative metrics, 15 reconstructions for each of the nine phantom setups were selected, yielding 135 image sets for evaluation. Individual readers used a digital form to record their evaluations, with images presented in random order. Sensitivity, specificity and accuracy were calculated from these scores. The confidence ratings were assigned numerical values (Table 2), and the mean confidence scores for true positives, true negatives, false positives and false negatives were calculated. In addition, the confidence ratings were summed (with positive values denoting correct evaluations, and negative values incorrect calls) to provide an estimation of accuracy and confidence combined (e.g., a true positive result, with a ‘completely confident’ rating would be considered + 4, whereas a false positive call with the same confidence rating would be scored as –4). There were nine phantom setups, and each side of the pituitary gland phantom could be true or false such that the maximum and minimum scores for each reconstruction were 72 and -72, respectively. To compare the reconstruction types, the average reviewer scores were calculated.

**Table 2:** Likert scales and numerical values

Confidence rating	Numerical values	
	True	False
Not confident at all	0	0
Slightly confident	+ 1	− 1
Somewhat confident	+ 2	− 2
Fairly confident	+ 3	− 3
Completely confident	+ 4	− 4

## Results

### Imaging

While imaging the phantom the scanner had a count rate of 253 kcps, a dead-time factor of approximately 1.07 (where 1.00 would be zero dead-time) and a scatter fraction of approximately 0.31 (where 0 would indicate no scatter). In comparison, clinical Met-PET studies have a count rate of approximately 170 kcps, dead-time factors around 1.03 and scatter fractions of approximately 0.25. These results indicate that the phantom’s overall count rate and proportion of scatter, while similar to clinical studies, are slightly higher and would actually make the detection of the lesion in the phantom more challenging.

The activity concentrations in the cerebellum measured in the reconstructed images were approximately 2.7 kBq/ml, which are comparable to those in clinical images of 2.4 to 6.7 kBq/ml. The pituitary region of the normal phantom had approximately 2.7 times the concentration of the cerebellum (7.4 kBq/ml) with the tumor phantoms exhibiting higher concentrations depending on the size and activity of the tumors. Importantly, both regions have activity concentrations that are comparable to clinical imaging albeit at the lower end of the range seen in clinical practice. This situation represents a 'worse-case' scenario and will hopefully ensure the finding of this work are applicable to the full range of situations seen in clinical practice.

### Measurements of radioactive concentrations

The radioactive concentration decay corrected to the start of the imaging in the pituitary gland was measured to be 19.1 kBq/ml (96% of 20 kBq/ml target). The radioactive concentrations of the tumors were found to be 42.5, 99.6 and 142.8 kBq/ml generating ratios of 2.2, 5.2 and 7.5 to pituitary gland (expected values 2.0, 5.0 and 8.0, respectively). For each resin mixture, the coefficient of variation in the CPM in the four cylinders was ≤ 0.6%.

### Measurements of Hounsfield units

**Fig. 4:**
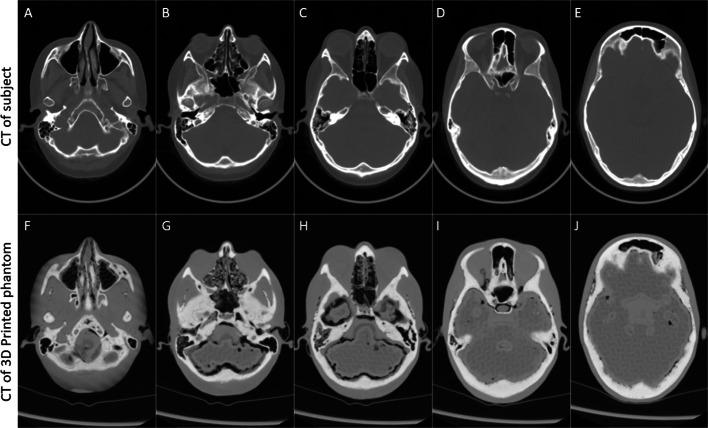
Comparison of CT scans from the subject and the 3D printed phantom. (**A–E**), axial slices from the patient’s original CT scan that were used to create the phantom; (**F–J**), matched axial slices from the CT scan of the 3D printed phantom

The mean Hounsfield units from CT scans obtained from the patient and the 3D printed phantom were 580 and 623 HU, respectively, for the bone region, and 30 and 122 HU, respectively, for the soft tissue region. Importantly, the appearance of the two CT scans was very similar, except that the phantom provided less detail (e.g., in distinguishing cortical and cancellous bone), and air gaps were visible where the two parts of the phantom were joined (Fig. 4).

### Reconstructed maxima

The reconstructed maxima were generally highest using OSEM with PSF and TOF with a 1-mm Gaussian filter. As expected, the maxima decreased as the Gaussian filter was increased to 2 and 3 mm. When compared to otherwise identical reconstructions, the use of PSF and TOF corrections both increased the maxima. The highest maxima from the BPL reconstructions were observed at the lowest Beta values (Additional file 1: Table 1).

### Tumor-to-pituitary contrast

The tumor-to-pituitary contrast was generally highest for OSEM reconstructions with PSF and TOF at seven to nine iterations. Importantly, when compared to otherwise identical reconstructions, the use of TOF markedly increased contrast. Contrast also increased as the BPL Beta value decreased.

The normal pituitary phantom was best visualized at low numbers of OSEM iterations and the highest beta values for the BPL reconstructions (Additional file 1: Table 2).

### Reconstructed noise

Noise in the reconstructed images was generally lowest using the BPL reconstruction with Beta values above 300. Noise increased as the number of OSEM iterations increased and decreased as the Gaussian filter was increased from 1 to 3 mm. PSF and TOF corrections had little discernible effect on noise (Additional file 1: Table 3).

**Table 3:** Results of image evaluation

Reconstruction	Confusion matrix			Confidence				Combined
	Sensitivity	Specificity	Accuracy	True positive confidence	True negative confidence	False positive confidence	True negative confidence	Summed confidence
OSEM 128 TOF 3i24s	62	95	83	1.86	2.86	− 0.25	− 1.59	30.8
OSEM 128 TOF PSF 3i24s	78	90	89	2.78	2.32	− 0.88	− 0.44	37.1
OSEM 256 TOF PSF 3i24s	78	90	88	3.02	2.51	− 1.38	− 1.22	39.1
OSEM 256 TOF PSF 5i24s	81	79	84	3.09	2.30	− 1.47	− 2.67	34.4
OSEM 256 TOF PSF 7i24s	84	60	74	3.01	1.98	− 0.84	− 1.00	30.1
OSEM 256 TOF PSF 9i25s	86	56	73	2.94	2.04	− 0.94	− 1.00	29.1
BPL 256 TOF PSF *β*50	86	32	60	3.03	1.31	− 1.18	− 4.00	19.2
BPL 256 TOF PSF *β*100	89	47	70	3.14	1.79	− 1.12	0.00	27.3
BPL 256 TOF PSF *β*200	86	74	84	3.16	2.24	− 0.95	0.00	37.3
BPL 256 TOF PSF *β*300	83	84	88	3.07	2.52	− 1.54	0.00	39.7
BPL 256 TOF PSF *β*400	77	93	90	2.98	2.51	− 1.33	− 0.90	39.6
BPL 256 TOF PSF *β*500	68	95	86	3.02	2.86	− 0.25	− 1.65	39.7
BPL 256 TOF PSF *β*600	68	95	86	2.93	2.77	− 1.25	− 1.76	37.7
BPL 256 TOF PSF *β*800	67	95	86	2.83	2.82	− 1.00	− 1.72	37.3
BPL 256 TOF PSF *β*1000	64	95	86	2.71	2.86	− 1.00	− 1.75	37.1

### Time of flight

**Fig. 5:**
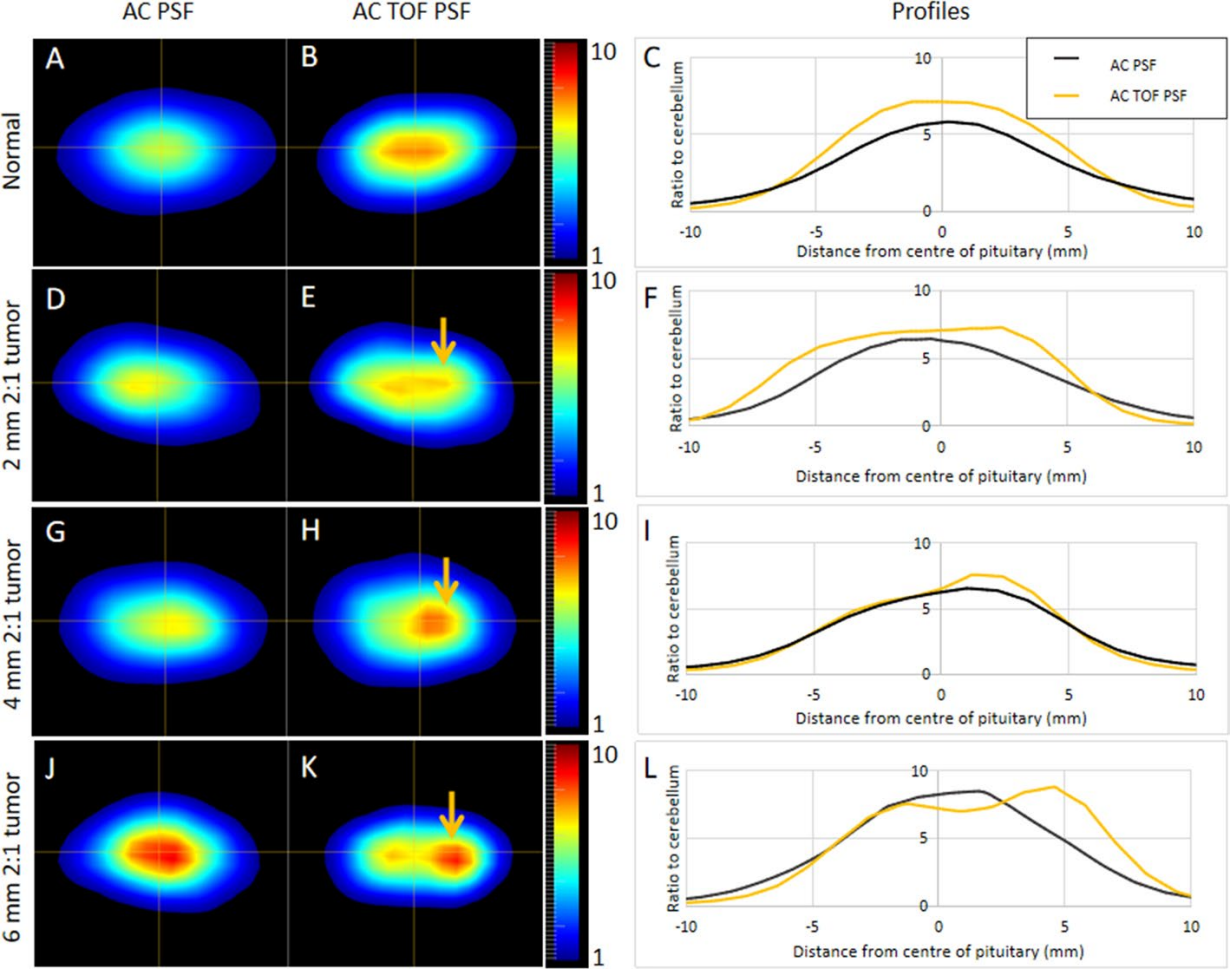
Impact of TOF on reconstructed images. Representative images and line profiles for the normal pituitary phantom (**A**–**C**) and for phantoms containing tumors of different diameters with an activity ratio of 2:1 compared with the background gland (**D–F**, 2 mm; **G–I**, 4 mm; **J–L**, 6 mm). Key: AC, attenuation correction; PSF, point spread function; TOF, time of flight

In all phantom setups, a comparison between OSEM reconstruction with and without TOF was performed using a 256 × 256 matrix, 2-mm Gaussian filter, and three iterations with 24 subsets. For each setup, the maximum signal was increased when using TOF. Importantly, in all pituitary phantoms harboring tumors, the tumor-to-pituitary contrast was increased with TOF. Indeed, reconstructions without TOF returned the lowest contrast in seven of the eight phantoms containing a tumor (Additional file 1: Table 2). Figure 5 shows some example images and line profiles for the normal, 2 mm 2:1, 4 mm 2:1 and 6 mm 2:1 phantom setups.

### Point spread function correction

**Fig. 6:**
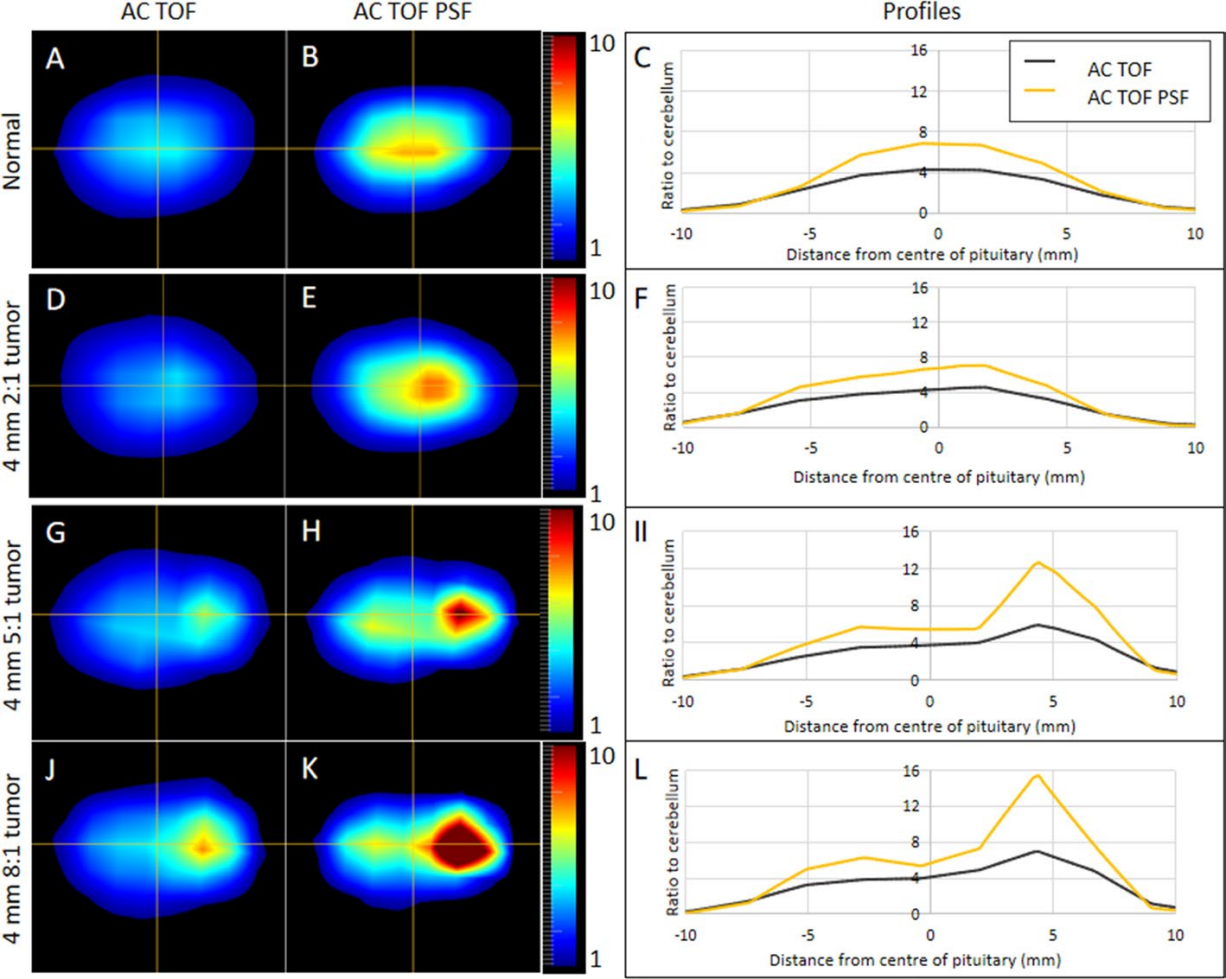
Point spread function correction analysis. (**A**, **B**, **D**, **E**, **G**, **H**, **J** and **K**) are the central slices from the normal pituitary gland phantom (**A**, **B**), 2:1 ratio 4-mm tumor (**D**, **E**), 5:1 ratio 4-mm tumor (**G**, **H**) and 10:1 ratio 4-mm tumor (**J**, **K**) reconstructed with attenuation correction and time-of-flight (AC TOF) (**A**, **D**, **G**, **J**) and with attenuation correction**,** time of flight and PSF (AC TOF PSF) (**B**, **E**, **H**, **K**). Profiles along the horizontal line are shown in panels (**C**, **F**, **I** and **L**) with black line representing the AC TOF reconstructions and the yellow line representing the AC TOF PSF reconstructions

In all phantom setups, the use of point spread function correction (PSF [GE SharpIR]) increased both signal and noise (Additional file 1: Tables 1 and 3). In the normal phantom, the symmetrical appearance was preserved after applying PSF; this can be appreciated in Fig. 6 (panels A–C). The tumor-to-background contrast was improved in seven of the eight tumor-containing phantoms (Additional file 1: Table 2). However, the noise was higher when using PSF in seven of nine phantoms, but the same in two. Figure 6 shows example images and profiles for the normal phantom and the 4-mm tumors with tumor-to-pituitary ratios of 2:1, 5:1 and 8:1, respectively.

### Matrix size (pixel size)

**Fig. 7:**
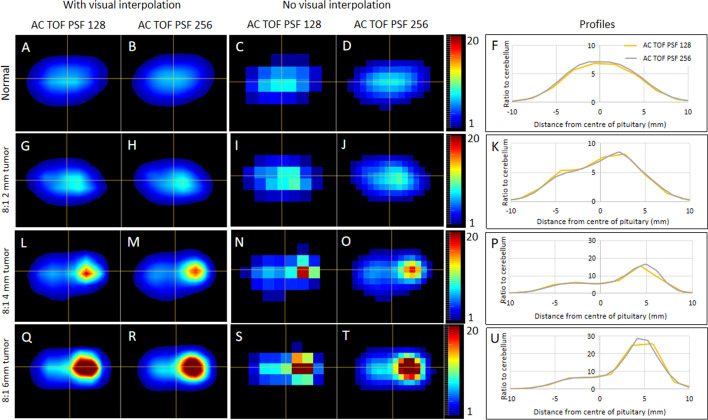
Matrix size analysis. The normal pituitary phantom (**A–E**) and the pituitary tumor phantoms with an 8:1 ratio (2 mm—**G–K**, 4 mm—**L–P** and 6 mm—**Q–U**) are shown following reconstruction using a 128 × 128 matrix size (columns labeled ‘AC TOF PSF 128’) and using a 256 × 256 matrix size (columns labeled ‘AC TOF PSF 256’). To highlight the effect the images are displayed with and without visual interpolation (uppermost column headings). The signal profiles along the horizontal cross-hair are shown in the column labeled profiles. The 128 × 128 and 256 × 256 matrix profiles are shown as yellow and gray lines, respectively

The use of smaller voxels increased the maximum voxel values in eight out of nine of the tumor phantoms. This is due to the partial volume effect having less influence when the pixels are smaller. A consequence of using smaller pixels is that each voxel has less data in it. However, the noise was lower in six out of the nine phantom setups when using smaller voxels and the same in the other three. As shown in Fig. 6, in addition to a marginal improvement in the maximum signal, having smaller voxels effectively means that we are sampling four times as much, and consequently, the images without visual interpolation (Fig. 7C, D, I, J, N, O, S, T) look smoother and more representative of the known distribution. The same effect (although to a lesser extent) can also be seen with the visual interpolation (Fig. 7A, B, G, H, L, M, Q, R). The contrast was improved in four out of the eight pituitary tumor phantom setups and worse in the other 4.

### OSEM smoothing and iterative reconstruction updates

**Fig. 8:**
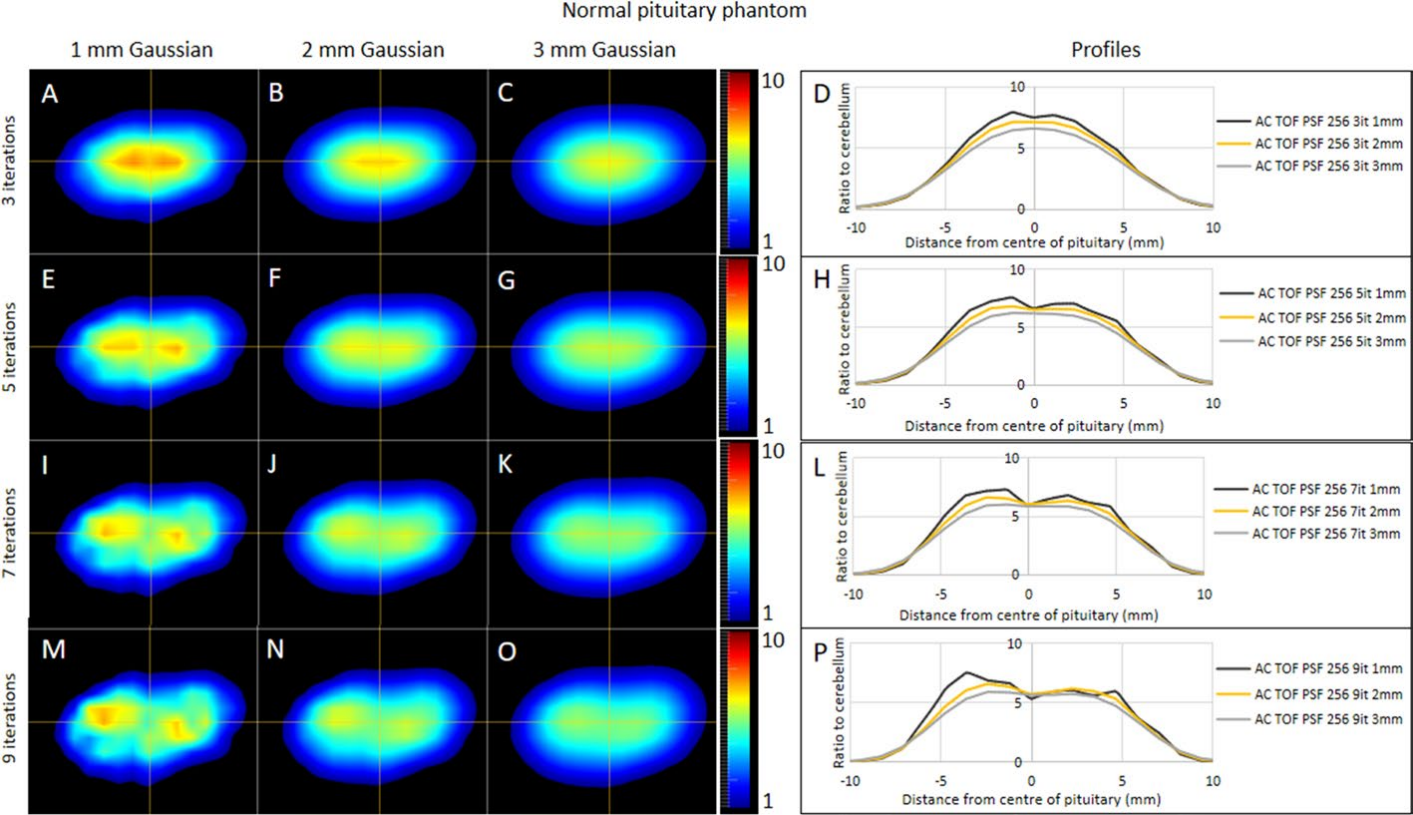
Number of OSEM updates and Gaussian filtering analysis using the normal pituitary gland phantom. The normal pituitary gland phantom was reconstructed using 1-, 2- and 3-mm post-reconstruction Gaussian filters and 3, 5, 7 and 9 numbers of OSEM iterations all with 24 subsets. The central slice of the resulting 12 images are displayed in panels (**A**–**C**, **E**–**G**, **I**–**K** and **M**–**O**). The signal along with the horizontal part of the cross-hairs is shown as profiles in panels (**D**, **H**, **L** and **P**)

**Fig. 9:**
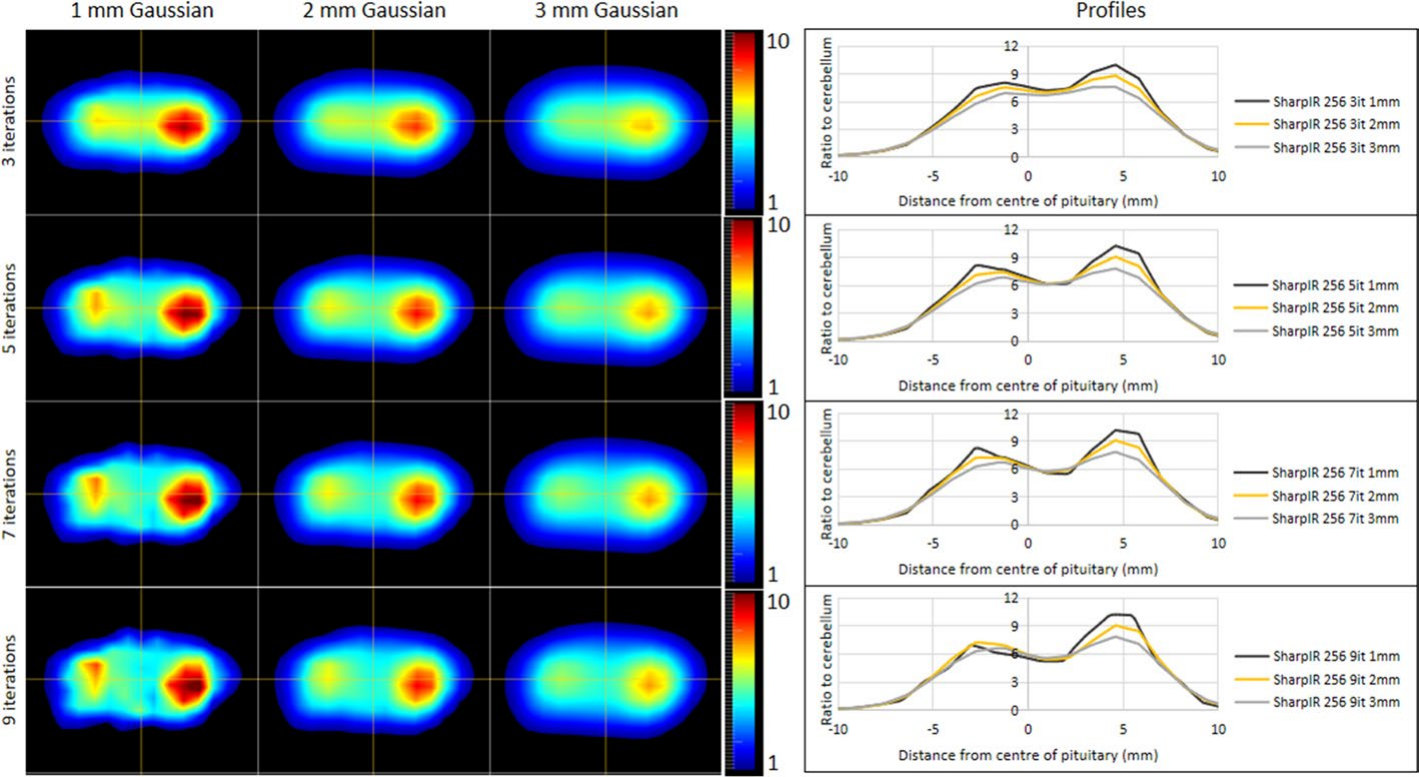
Number of OSEM updates and Gaussian filtering analysis example using the 6-mm 2-to-1 ratio pituitary tumor phantom. The 6-mm pituitary tumor phantom with a 2-to-1 ratio of activity concentration was reconstructed using 1-, 2- and 3-mm post-reconstruction Gaussian filters and 3, 5, 7 and 9 numbers of OSEM iterations all with 24 subsets. The central slice of the resulting 12 images is displayed in panels (**A**–**C**, **E**–**G**, **I**–**K** and **M**–**O**). The signal along with the horizontal part of the cross-hairs is shown as profiles in panels (**D**, **H**, **L** and **P**)

As expected, as the post-reconstruction Gaussian filtering FWHM was changed from 1 to 3 mm, the maximum signal for each phantom was reduced and the noise decreased. Overall, the contrast was highest when the Gaussian filter FWHM was set to 1 mm but the high levels of noise caused erroneous foci of signal (panels E, I and M in Figs. 8 and 9). The 2-mm Gaussian filter had lower contrast than the 1-mm Gaussian filter and importantly demonstrated lower noise with fewer false positive foci. The 3-mm Gaussian filter produced images which most closely approximated the expected appearance of a normal pituitary gland (Fig. 8), but was associated with reduced contrast between the different tumors and the background pituitary gland (Fig. 9). Therefore, the 2-mm Gaussian was chosen as the optimal option.

In some phantom setups, the maximum signal increased with the number of iterations. However, a consequence of this was an increase in the noise. An example of this can be seen in Fig. 8 panel M where there appear to be two discrete foci that could be interpreted as two areas of interest in a clinical scan. This appearance can still be appreciated when using the 2-mm Gaussian filter at nine iterations (Fig. 8N).

### Bayesian penalized likelihood reconstruction

**Fig. 10:**
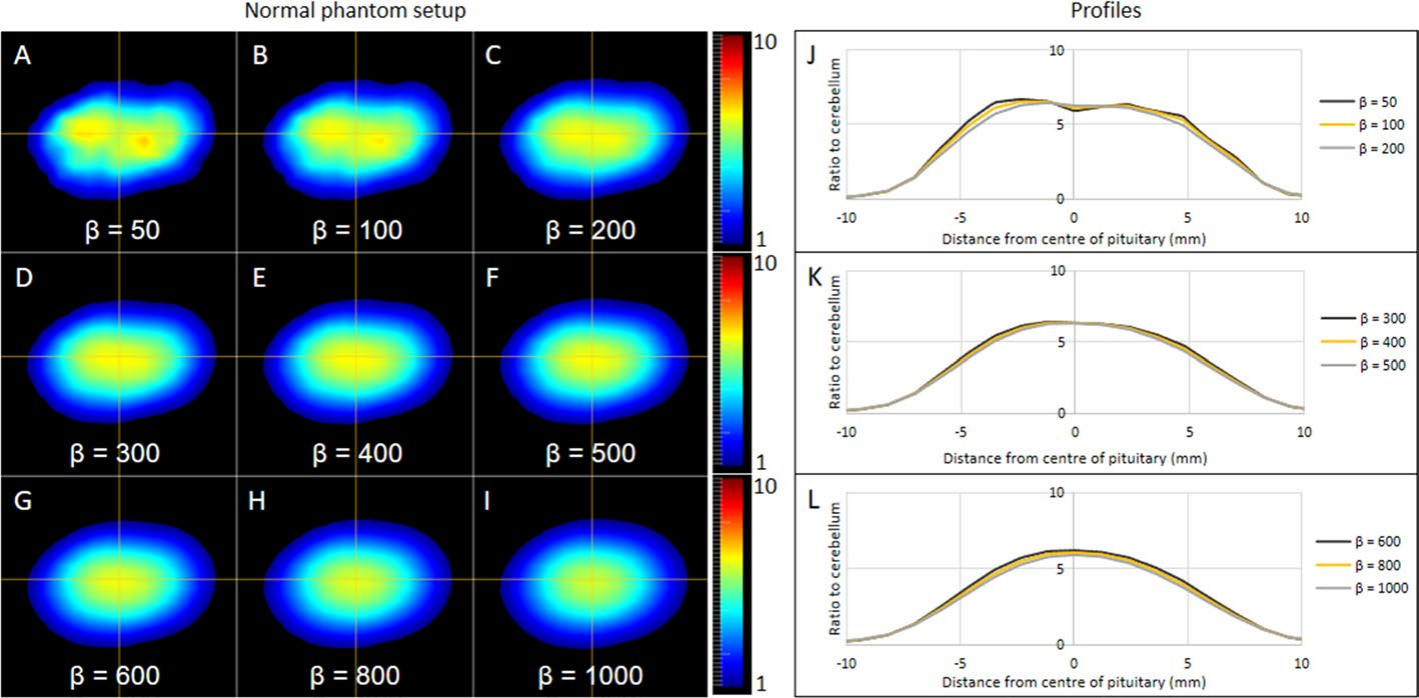
BPL (Q.Clear) reconstructions of the normal pituitary phantom. The normal pituitary phantom was reconstructed using the BPL algorithm with a range of *β* values from 50 to 1000. Panels **A** to **I** show the central slice of the normal pituitary phantom with *β* values of 50, 100, 200, 300, 400, 500, 600, 800, 1000, respectively. Profiles plotted along the horizontal cross-hairs are shown in panels (**J** to **L**)

**Fig. 11:**
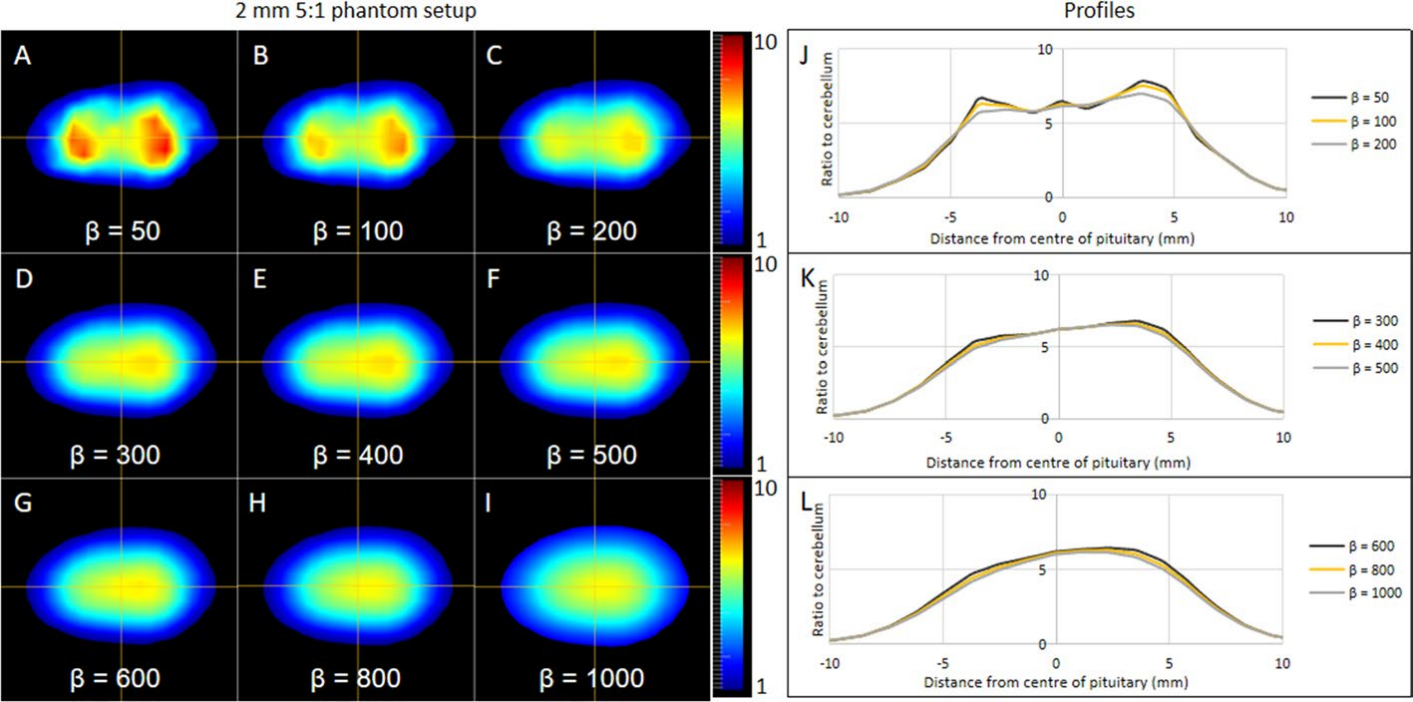
BPL (Q.Clear) reconstructions of 2-mm 5:1 pituitary tumor phantom. The 2-mm 5:1 pituitary tumor phantom was reconstructed using the BPL algorithm with a range of *β* values from 50 to 1000. Panels **A** to **I** show the central slice of the normal pituitary phantom with *β* values of 50, 100, 200, 300, 400, 500, 600, 800, 1000, respectively. Profiles plotted along the horizontal cross-hairs are shown in panels (**J** to **L**)

The BPL algorithm was used with the same 256 × 256 matrix size, including PSF and TOF; therefore, the only parameter that required optimization was the regularization parameter (*β*). Figure 10 shows an example set of images for the normal pituitary phantom, and Fig. 11 shows an example set of images for the 5:1 2-mm pituitary phantom; both figures show the effect of varying the *β* value. Quantitatively, the maximum signal for each phantom setup was highest when deploying a *β* value of 50 and reduced as the *β* value increased. For the noise measured in the background cerebellum, the lowest measurements in each phantom setup were observed when using the highest *β* value of 1000. The noise increased as the *β* values decreased and were highest at a *β* value of 50. Left to right contrast was highest in seven of the eight pituitary tumor phantoms when *β* = 50 except for 2 mm 8:1 where the maximum contrast was at *β* = 200; this result can be attributed to noise on the opposite side of the pituitary that was almost as high as the true signal.

### Expert image evaluation

Of the 26 reconstructions used in the preceding quantitative analysis 15 were chosen to be evaluated by the group of expert reviewers. These included six OSEM reconstructions and nine BPL reconstructions; see Table 1 for the full list of reconstructions used for the expert image evaluation.

The expert evaluations revealed that sensitivity and accuracy were improved by using PSF correction, whereas specificity was higher without PSF correction. Both the true positive and false positive confidence were higher when using PSF correction, indicating that although the confidence in which a reader is identifying true tumors is improved by PSF correction, they are also more confident reporting tumors that are not present.

There was no difference in the sensitivity or specificity between the two pixel sizes. The 128 × 128 matrix size demonstrated slightly improved accuracy , while the 256 × 256 matrix size yielded a higher combined confidence score. The 256 × 256 matrix had higher confidence ratings for true positive, false positive, true negative and false negative.

The expert evaluations indicate that specificity and accuracy were highest for three iterations 24 subsets but sensitivity increased with the iterative updates and was highest for nine iterations 24 subsets. The reconstruction using five iterations 24 subsets had the highest mean confidence ratings for true positives, while the three iterations 24 subset had the highest true negative rating.

Furthermore, sensitivity was highest for *β* = 100, specificity was highest for *β* = 1000, and overall accuracy was highest for *β* = 400. The highest mean confidence for true positives was when *β* = 200, and the highest mean confidence for false negatives were at *β* = 500 and 1000. The combined accuracy and confidence results were highest for *β* = 300.

### Application of optimized parameters

**Fig. 12:**
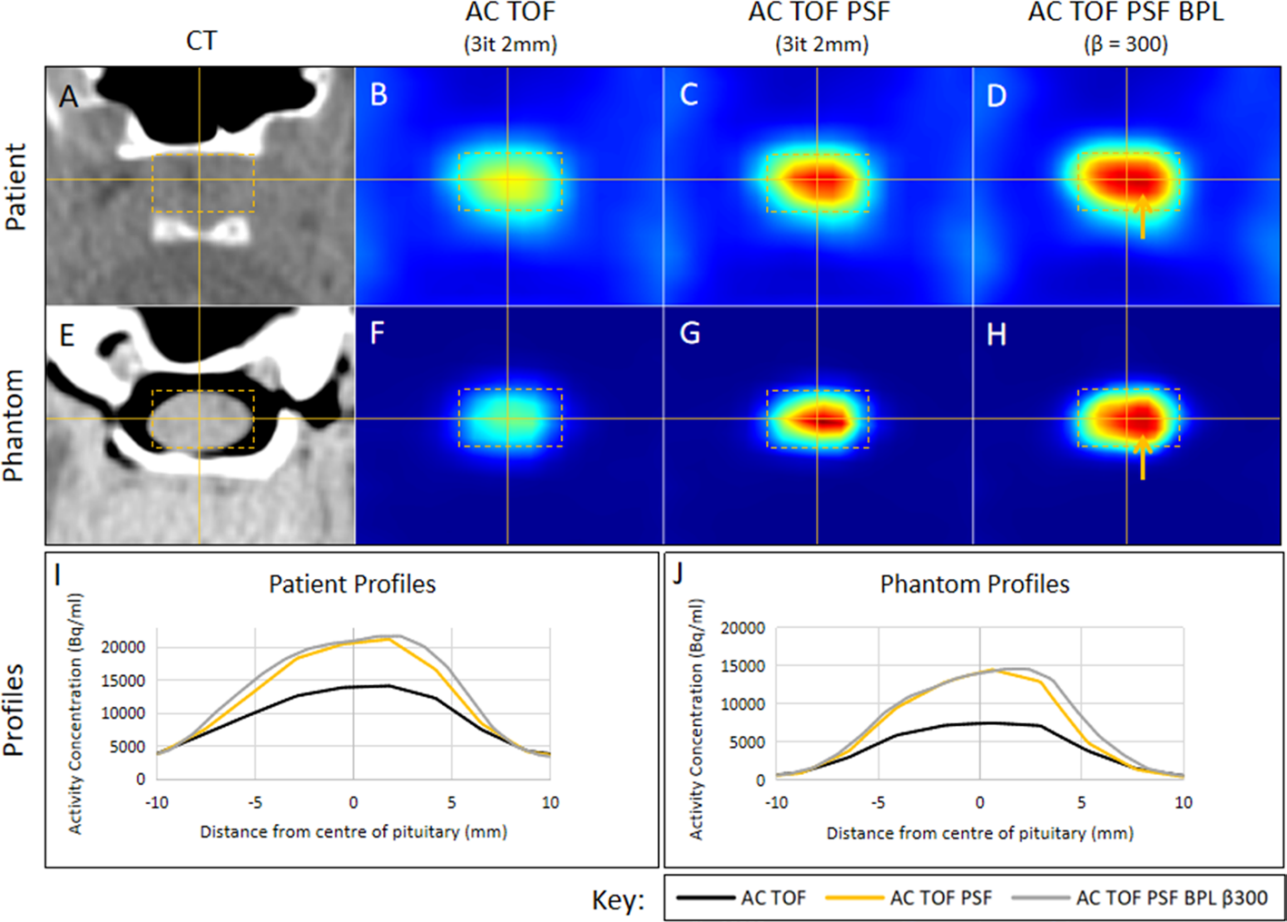
Comparison of phantom images with actual clinical images derived from the patient on whom the phantom was modeled. Images from the patient are shown next to the 2-mm 5:1 pituitary tumor phantom. Panels **A** and **E** show the CT images of the patient and phantom, respectively, the cross-hairs indicate the center of the pituitary gland, and the yellow box demarcates the area of interest. Panels **B** and **F** show the PET images of the patient and phantom that were reconstructed using OSEM with three iterations, 24 subsets, attenuation correction, time-of-flight, a 2-mm Gaussian filter without point-spread-function correction (PSF). Panels **C** and **G** show the PET images of the patient and phantom that were reconstructed in the same way but using PSF. Panels **D** and **H** show the PET images of the patient and phantom that were reconstructed using the optimized BPL reconstruction using a *β* value of 300 (PSF). The orange arrows correspond to the location in the gland where a 2-mm pituitary tumor was subsequently removed at surgery

The clinical Met-PET images of the same patient used to create the phantom were chosen to compare the optimized reconstruction parameters (AC, TOF PSF BPL with *β* = 300) to the previously used reconstructions (AC TOF 3 iterations 24 subsets and AC TOF PSF 3 iterations 24 subsets). Figure 12 shows the same three reconstructions for the patient and for the 5:1 2 mm tumor phantom. It can be seen that qualitatively the images look similar and the same improvements can be seen when PSF is applied and when using BPL. The profiles show that the activity concentrations in the patient and the phantom are similar, although the phantom has slightly lower concentrations.

## Discussion

We have demonstrated for the first time that a phantom which is capable of mimicking differential radiotracer uptake by normal and tumoral pituitary tissue can be created using radioactive 3D printing. Importantly, the model allowed the phantom tumors to be placed in direct contact with the phantom normal gland, thereby replicating the situation found in patients where tumors are often embedded within the normal gland. In addition, we housed these pituitary gland phantoms in a MEX 3D printed skull that approximated the attenuation properties of surrounding bone and soft tissue. Using this bespoke phantom, we were able to optimize parameters for pituitary gland imaging using a well-defined ground truth.

Three-dimensional printing of the pituitary gland and tumors using radioactive resin (Fig. 1) has a number of advantages over traditional fluid-filled phantoms. In particular, the absence of an inactive boundary (between the pituitary gland and tumor) improved our confidence when optimizing image parameters beyond what is possible using patient data alone (where the radioactive concentrations are unknown). Another advantage is that filling a sphere with radioactive liquid is difficult when using very small volumes (e.g., 34 μl for a sphere 2-mm in diameter), and is coupled with the potential for unwanted elongation of the sphere (along the filling tube). Moreover, filling small volumes is more challenging when using liquids with a high surface tension (such as water) because air may become trapped.

A potential disadvantage of radioactive 3D printing is occasional printing failure as experienced in this study, where one of the nine phantoms (5:1 6-mm lesion) did not print. This is a recognized issue with 3D printing and can, to some degree, be overcome by printing multiples of objects in different locations on the build plate.

At our center, PET imaging of the pituitary gland is usually performed using ^11^C-methionine, and therefore, the use of ^18^F in the phantom could have had an effect on the results. The reason for this is because ^18^F has a shorter mean positron range of 0.6 mm in water compared to ^11^C, which has a mean range of 1.2 mm [20]. However, the very short half-life of ^11^C (20 min) made it impractical to use this radiotracer in this work due to the large amounts of activity that would have been required to enable the same radioactive 3D printing.

Creating the phantom using patient images was relatively simple and the methodology described here (Fig. 2) could be applied to other similar phantoms (e.g., a small parathyroid adenoma embedded in the thyroid gland). A further improvement might be to split the phantom in a non-horizontal plane, thus avoiding the creation of an air gap which correlates with just one or two slices of the CT. Printing the surrounding anatomy using two materials was successful and enabled us to approximate the attenuation properties of the adjacent structures (Fig. 3). Other workers have recently demonstrated that the use of alternative materials (such as wood filled filaments) may provide attenuation properties that even more closely approximate those of soft tissues [21].

The Prusa MMU2 uses one nozzle and switches between materials by automatically extracting the filament and loading another; this is known as a tool change. During the printing of the phantom, almost every layer contained both materials and therefore approximately every other layer required a tool change. Accordingly, there were approximately 100 tool changes during the printing of each half of the phantom and, due to the complex nature of these changes, these accounted for the majority of the printing errors which occurred during the early phase of the project. Most of these errors could be rectified and the print could continue, but in some cases they necessitated abandoning the printing. In the majority of cases, the error occurred while the concrete-filled filament was either being loaded or unloaded, reflecting the brittle nature of the concrete-filled filament. Two changes to the printing setup reduced the frequency of these errors: firstly, ensuring the concrete filament was very dry and, secondly, that the tension on the filament gears was not too high. An alternative to the MMU2 would be a 3D printer that had two or more separate extruders.

Material extrusion 3D printing is commonly done using an infill percentage of approximately 20%; therefore, 80% of the internal volume is empty. This saves material, lowers costs and shortens print times significantly. However, a large proportion of our phantom required printing solid (effectively an infill of 100%) and therefore used a large amount of filament and was time-intensive. Using the default printer nozzle diameter of 0.4 mm and a layer height of 0.2 mm, each half of the phantom took approximately 60 h to print. These settings are optimized for printing aesthetically pleasing objects (e.g., 3D printing of a pituitary gland containing a tumor for educational and surgical planning purposes) [22]. Therefore, we elected to use a 0.8-mm nozzle and a layer height of 0.4 mm. These changes reduced the printing times for each half of the phantom to around 24 h because more material could be laid down per unit of time and, because there were fewer layers, there were also fewer tool changes. However, one disadvantage of printing with thicker layers is that making watertight printed objects is more challenging. As such, the final object had to be sealed after printing and a gel was used instead of water to mitigate the risk of leaking from the phantom.

Blinded review of the images was carried out using a purpose-built web-based tool (Fig. 3), which allowed us to assess both accuracy and confidence for each reconstruction type. In a clinical context, confidence when interpreting and reporting Met-PET is an important factor in key decision-making, e.g., when considering whether repeat surgery should be offered to a patient who has persistent disease despite primary surgery. Overall the confidence ratings for true positives were higher than for true negatives and, importantly, than for false positives and false negatives (Table 3). This finding likely reflects the way Met-PET is most commonly deployed in clinical practice, where it is used as a tool to confirm that a suspicious area on MRI is indeed the site of active disease; however, equally importantly, the false positive rate must be kept low so as to avoid recommendations for inappropriate surgery. Accordingly, a balance must be struck between the highest sensitivity without a detrimentally higher false positive rate, especially one with a high confidence rating. The optimal reconstructions for sensitivity, specificity and accuracy were all different. As anticipated, the highest sensitivity and highest specificity reconstructions were at the highest and lowest noise levels, respectively. The optimal combination of accuracy and confidence was achieved with BPL reconstructions and *β* values of 300, 400 and 500. Combining the metrics in this way, ensured that reconstructions which performed well but had low confidence would not score as highly as true calls that were made with confidence. Confidence ratings were recorded on a scale from 0 to 4 thus ensuring that “not confident at all” ratings did not contribute to the combined score, i.e., the nature of the call was not relevant when the call was made with no confidence.

The implementation of TOF made an important difference in the visual appearance of the images as well as quantitatively (Fig. 4). In some cases, tumors were only visible when TOF information was used. The most likely reason for this is that the OSEM reconstruction converges more quickly when the TOF information is included. The observed differences suggest that using TOF is an important factor when imaging the pituitary gland, especially when tumors are small. However, in systems without TOF capabilities this deficiency may be partly overcome by allowing the reconstruction algorithm to converge more fully. However, as we have demonstrated here, noise increases with the number of iterative reconstruction updates.

The use of point spread function correction increased signal in the tumor but did not have detrimental effects on the appearance of the normal pituitary gland without a tumor (Fig. 5). The profiles that can be seen in Fig. 5 highlight that PSF could make an important contribution in clinical cases where a tumor is not seen clearly.

The two voxel sizes examined in this work yielded similar findings: qualitatively, especially with visual interpolation, only a subtle difference was observed; quantitatively, the smaller voxel size increased maximum signal and contrast (reflecting the partial volume effect having less influence). Importantly, noise was not increased by the smaller voxels. Therefore, based on these marginal improvements, the smaller voxel size has been adopted for use in clinical practice in our center. Another area of potential improvement might be to use smaller slice intervals. Unfortunately, the scanner was unable to reconstruct in thinner slices, which precluded such comparison, but if the same pattern was seen as with the voxel size then this may also improve signal and contrast. Caution would need to be applied here though, as the levels of noise may become problematic if the voxels were made smaller again.

The use of a post-reconstruction filter is ubiquitous when using OSEM and its effect can be significant. It is therefore crucial to optimize the parameters of the filter to achieve a compromise between images that are too noisy or too smooth. In this study, we used three Gaussian filters with FWHMs of 1, 2 and 3 mm. The 1-mm filter did not smooth the noise sufficiently to be used clinically (Figs. 5 and 6), as non-tumoral areas that might be considered potential sites of increased tracer uptake could be seen. This appearance would be particularly problematic if encountered during optimization performed using datasets from patients or healthy volunteers because of the difficulty in establishing the veracity of such a finding without recourse to potentially inappropriate surgery. However, using the phantom, the nature of this positive signal is not in doubt (i.e., a false positive finding) and accordingly the 1-mm Gaussian filter would present challenges if adopted in clinical practice. On the other hand, while the 3-mm Gaussian filter was less likely to generate false positive results, the maximum signal over the tumor was notably diminished. Accordingly, a 2-mm Gaussian filter which balanced signal and noise was preferred.

We also investigated the impact of the number of OSEM iterations, comparing 3, 5, 7 and 9, each with 24 subsets. Signal and contrast were enhanced with increasing iterations; however, noise also increased. Despite this, increasing the number of iterations may potentially augment the detection of small tumors, and therefore, further work is required to explore the potential benefits and limitations of such an approach in clinical practice. For now, based on the preliminary findings reported here, the use of three iterations and 24 subsets is recommended to achieve both accuracy and confidence (Table 3).

The BPL algorithm gave rise to a wide range of results depending on the *β* values. When the *β* value was low, the signal was highest, but so was the noise. For some phantom setups, the lowest *β* value gave the highest contrast but, when compared with other *β* values in the blinded evaluation, also generated the highest false positive rate. A potentially important observation at low *β* values (and which was also noted at higher OSEM iterations) was the erroneous impression of increased tracer activity at the lateral margins of the pituitary phantom, (which were reported as potential tumor sites by several of the expert readers), and which is best appreciated in Figs. 10 and 11. The observed visual anomalies may be linked to edge artifacts, a type of distortion caused by reconstructed images that incorporate PSF correction, commonly referred to as Gibbs artifacts [23]. For the expert evaluations, the highest sensitivities were all observed at low *β* values, with the highest seen when *β* = 100. Specificity was highest when *β* was highest (1000), while accuracy was highest when *β* = 400. Accordingly, and depending on the imaging task in hand, it may be prudent to consider using different reconstruction parameters for different tasks.

In clinical practice, the most common reasons for performing molecular imaging of the pituitary gland are: (1) to confirm/reveal the site of a small tumor when anatomical imaging (e.g., MRI) is indeterminate; (2) to accurately localize site(s) of residual/recurrent tumor following primary therapy when further potentially curative treatment (e.g., surgery, stereotactic radiosurgery) is being considered. In these contexts, it is critically important that the reconstruction does not produce false positive findings that are held to be true with a high degree of confidence. Therefore, those parameters with the highest summed confidence (which applied a negative weighting to the false calls) are likely to represent the optimum reconstructions for confidently identifying tumor sites that are appropriate for targeted intervention.

Our results indicate that BPL reconstruction with a *β* value of 400 has the highest accuracy and one of the highest summed confidence scores. Another important factor is the sensitivity, and this was highest when BPL reconstruction had a *β* value of 100. Importantly, for those without access to BPL reconstruction, the OSEM reconstruction with TOF, PSF correction and nine iterations and 24 subsets had almost as high sensitivity.

## Conclusions

Using radioactive 3D printing, we have created a bespoke pituitary phantom that can mimic the presence of a small tumor which is embedded in the normal pituitary gland. We have then used this well-defined ground truth to optimize PET reconstruction in molecular pituitary imaging, identifying the following preferred parameters: BPL reconstruction with TOF, PSF correction and a *β* value of 300; for small tumors (< 4 mm) that have low contrast (2:1 or 5:1) sensitivity may be improved by using a *β* value of 100.

## Supplementary Information

**Additional file 1. Table 1**. Maximum signal normalized to the region of the cerebellum for all phantom setups and reconstruction parameters. Each column is individually color coded to denote the lowest value for that phantom as red and the highest value as green. **Table 2**. Contrast for all phantoms and reconstructions. Each column is individually color coded to denote the lowest value for that phantom as red and the highest value as green, with the exception of the normal phantom column where values closest to 1 are shown in green and those furthest from 1 in red. **Table 3** Noise for all phantoms and reconstructions. Each column is individually color coded to denote the lowest value for that phantom as green and the highest value as red.

## Data Availability

The datasets used and/or analyzed during the current study are available from the corresponding author on reasonable request.

## Abbreviations

AC: Attenuation correction
BPL: Bayesian penalized likelihood
CoV: Coefficient of variation
CT: X-ray computed tomography
FOV: Field of view
FWHM: Full width half maximum
MEX: Material extrusion 3D printing
OSEM: Ordered subset expectation maximization
PET: Positron emission tomography
PSF: Point spread function
TOF: Time-of-flight
VOI: Volume of interest
VP: Vat photopolymerization 3D printing
