# Supplementary material for: Development of a bespoke phantom to optimize molecular PET imaging of pituitary tumors
Source: EJNMMI Phys. 2023 Jun 1;10:34. doi: 10.1186/s40658-023-00552-9 (PMC10234958; doi:10.1186/s40658-023-00552-9)

**Supplementary material**

**Supplementary Table 1**. Maximum signal normalized to the region of the cerebellum for all phantom setups and reconstruction parameters. Each column is individually color coded to denote the lowest value for that phantom as red and the highest value as green.


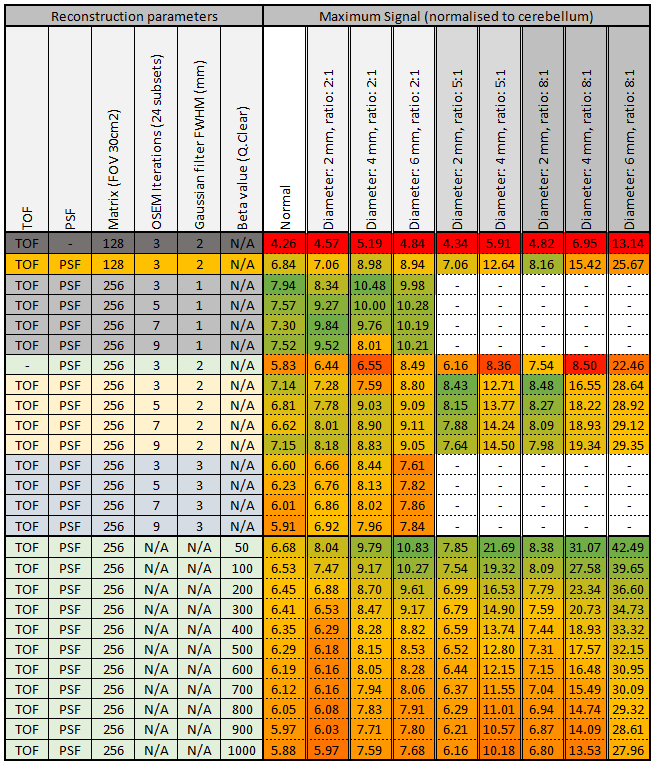


**Supplementary Table 2.** Contrast for all phantoms and reconstructions. Each column is individually color coded to denote the lowest value for that phantom as red and the highest value as green, with the exception of the normal phantom column where values closest to 1 are shown in green and those furthest from 1 in red.


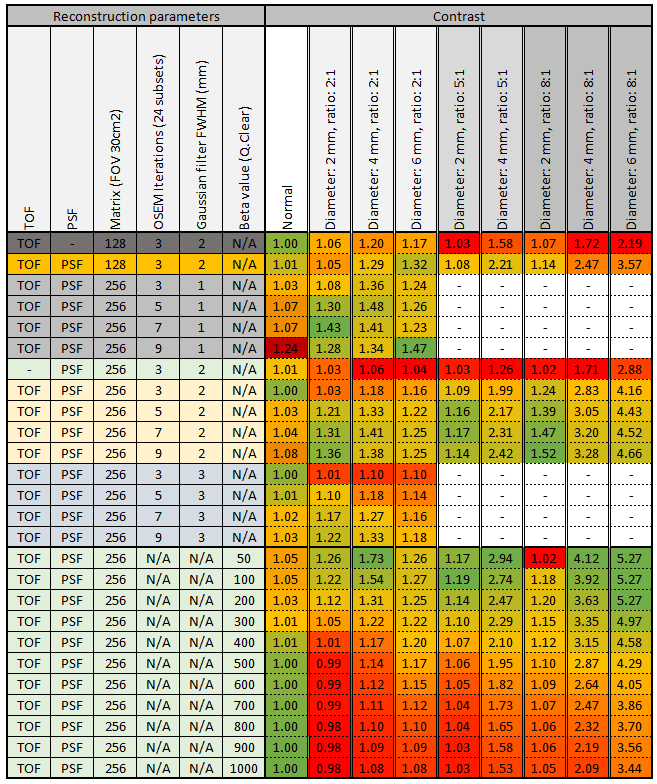


**Supplementary Table 3** - Noise (represented as coefficient of variation) for all phantoms and reconstructions. Each column is individually color coded to denote the lowest value for that phantom as green and the highest value as red.


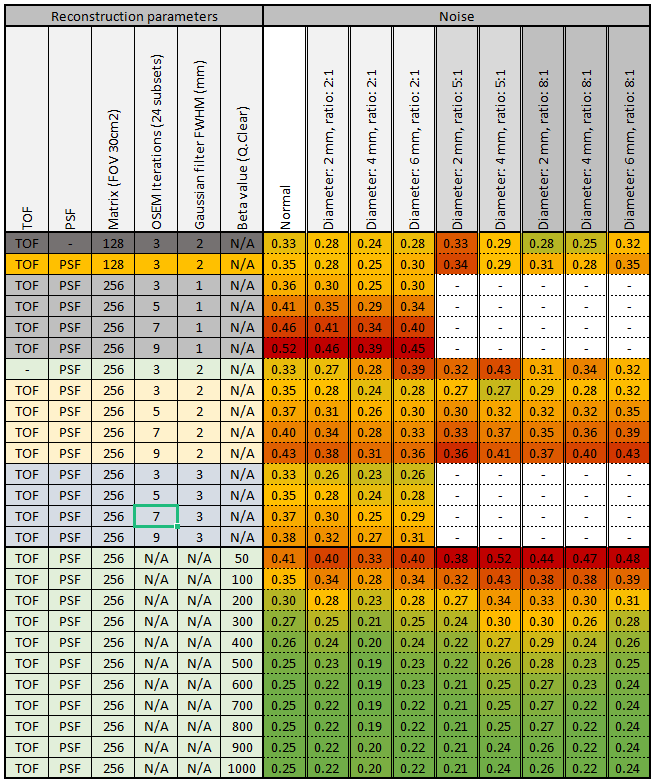

Supplement: Supplementary file 1 — Additional file 1. Table 1. Maximum signal normalized to the region of the cerebellum for all phantom setups and reconstruction parameters. Each column is individually color coded to denote the lowest value for that phantom as red and the highest value as green. Table 2. Contrast for all phantoms and reconstructions. Each column is individually color coded to denote the lowest value for that phantom as red and the highest value as green, with the exception of the normal phantom column where values closest to 1 are shown in green and those furthest from 1 in red. Table 3 Noise for all phantoms and reconstructions. Each column is individually color coded to denote the lowest value for that phantom as green and the highest value as red. [file 40658_2023_552_MOESM1_ESM.docx]
